# Meltrin β/ADAM19 Interacting with EphA4 in Developing Neural Cells Participates in Formation of the Neuromuscular Junction

**DOI:** 10.1371/journal.pone.0003322

**Published:** 2008-10-02

**Authors:** Norihiro Yumoto, Shuji Wakatsuki, Tomohiro Kurisaki, Yoshinobu Hara, Noriko Osumi, Jonas Frisén, Atsuko Sehara-Fujisawa

**Affiliations:** 1 Department of Growth Regulation, Institute for Frontier Medical Sciences, Kyoto University, Shogo-in, Kyoto, Japan; 2 Division of Developmental Neuroscience, Center for Translational and Advanced Animal Research (CTTAR), Tohoku University School of Medicine, Sendai, Japan; 3 Department of Cell and Molecular Biology, Medical Nobel Institute, Stockholm, Sweden; Duke Unviersity, United States of America

## Abstract

**Background:**

Development of the neuromuscular junction (NMJ) is initiated by the formation of postsynaptic specializations in the central zones of muscles, followed by the arrival of motor nerve terminals opposite the postsynaptic regions. The post- and presynaptic components are then stabilized and modified to form mature synapses. Roles of ADAM (A Disintegrin And Metalloprotease) family proteins in the formation of the NMJ have not been reported previously.

**Principal Findings:**

We report here that Meltrin β, ADAM19, participates in the formation of the NMJ. The zone of acetylcholine receptor α mRNA distribution was broader and excess sprouting of motor nerve terminals was more prominent in *meltrin β*–deficient than in wild-type embryonic diaphragms. A microarray analysis revealed that the preferential distribution of *ephrin-A5* mRNA in the synaptic region of muscles was aberrant in the *meltrin β*–deficient muscles. Excess sprouting of motor nerve terminals was also found in ephrin-A5 knockout mice, which lead us to investigate a possible link between Meltrin β and ephrin-A5-Eph signaling in the development of the NMJ. Meltrin β and EphA4 interacted with each other in developing motor neurons, and both of these proteins localized in the NMJ. Coexpression of Meltrin β and EphA4 strongly blocked vesicular internalization of ephrin-A5–EphA4 complexes without requiring the protease activity of Meltrin β, suggesting a regulatory role of Meltrin β in ephrin-A5-Eph signaling.

**Conclusion:**

Meltrin β plays a regulatory role in formation of the NMJ. The endocytosis of ephrin-Eph complexes is required for efficient contact-dependent repulsion between ephrin and Eph. We propose that Meltrin β stabilizes the interaction between ephrin-A5 and EphA4 by regulating endocytosis of the ephrinA5-EphA complex negatively, which would contribute to the fine-tuning of the NMJ during development.

## Introduction

A neuromuscular junction (NMJ) is the synapse between an axon terminal of a motor neuron on the presynaptic side and a specialized region of a muscle fiber on the postsynaptic side. Formation of the NMJ during development is a multistep process[1]–[3] . In the initial phase of NMJ formation, nicotinic acetylcholine receptors (AChRs) cluster with other postsynaptic proteins in the central regions of the myofibers after myogenesis independently of innervations by motor neurons; this process is called “prepatterning” [4]–[6]. Some proteins expressed in the muscle, MuSK (a muscle-specific receptor tyrosine kinase), LRP4, and Dok-7, are essential for this process [7]–[9]. The axons of motor neurons are guided by chemoattractants and chemorepellents to elongate toward target muscles so that the axon terminals can contact the prepatterned postsynaptic regions. The pre- and postsynaptic regions then differentiate and are stabilized and modified by the elimination of redundant synapses to form structurally and functionally mature NMJs. However, precisely how these processes occur remains an important unanswered question.

The ADAM (a disintegrin and metalloprotease) family proteins play critical roles in morphogenesis, fertilization, and diseases. Some ADAM proteins contain an active metalloprotease domain, and ADAM proteases modulate the ectodomain shedding of various membrane proteins, such as membrane-anchored growth factors, receptors, and adhesion molecules. For example, tumor necrosis factor-α converting enzyme (TACE/ADAM17) is essential for the phorbol ester–stimulated shedding of the ectodomains of various membrane-anchored proteins [10], and Kuzbanian/ADAM10 is involved in the ectodomain shedding of multiple substrates, including ephrin, Notch ligands, and cadherins [11]–[15].

Meltrin β, an ADAM protein that contains an active metalloprotease domain, is strongly expressed in the heart and peripheral nervous system during development [16], [17]. Meltrin β mediates the ectodomain shedding of neuregulin β1, a membrane-anchored ErbB ligand, in cultured cells [18]–[20].

Although the protease functions of ADAM proteins have been studied most intensively, these proteins contain other domains [21] such as the disintegrin domain, and it is important to investigate the functions that ADAM proteins may possess in addition to their protease activity. We previously generated *meltrin β^−/−^* mice to further study the properties of Meltrin β. Using these mice, we and another group showed that Meltrin β is involved in the heart development [22]–[24]. In the C57BL/6 background, *meltrin β* ^−/−^ mice are born, but die immediately after birth. Neonatal death is often caused by respiratory failure, including defective regulation of the contractions of respiratory muscles, such as the diaphragm and intercostal muscles. This finding, combined with the expression of Meltrin β in the peripheral nervous system [17], led us to further investigate the roles of this gene in the development of the NMJ.

In this study, we found that Meltrin β plays a regulatory role in the formation of NMJs. The zone of acetylcholine receptor α mRNA distribution was broader and excess sprouting of motor nerve terminals was more prominent in *meltrin β*–deficient than in wild-type embryonic diaphragms. A microarray analysis revealed aberrant distribution of ephrin-A5 in addition to acetylcholine receptor α transcripts in *meltrin β^−/−^* mice. Excess sprouting of axon terminals were also found in ephrin-A5^−/−^ mice, which lead us to examine whether Meltrin β regulates ephrin-A5-Eph signaling in the formation of the NMJ. Meltrin β interacted with EphA4 in motor neurons during development, and the expression of Meltrin β in EphA4-expressing cells blocked the vesicular internalization of ephrin-A5 bound to EphA4, independently of the Meltrin β metalloprotease function. Previous studies indicated the requirement of endocytosis in efficient contact-dependent repulsion induced by ephrin-Eph signaling. From these results and previous findings, we propose that Meltrin β negatively regulates ephrin-A5-EphA vesicular internalization, which could be one of mechanisms to stabilize the developing NMJ.

## Results

### Meltrin β is expressed at the NMJ and involved in its formation

**Figure 1:**
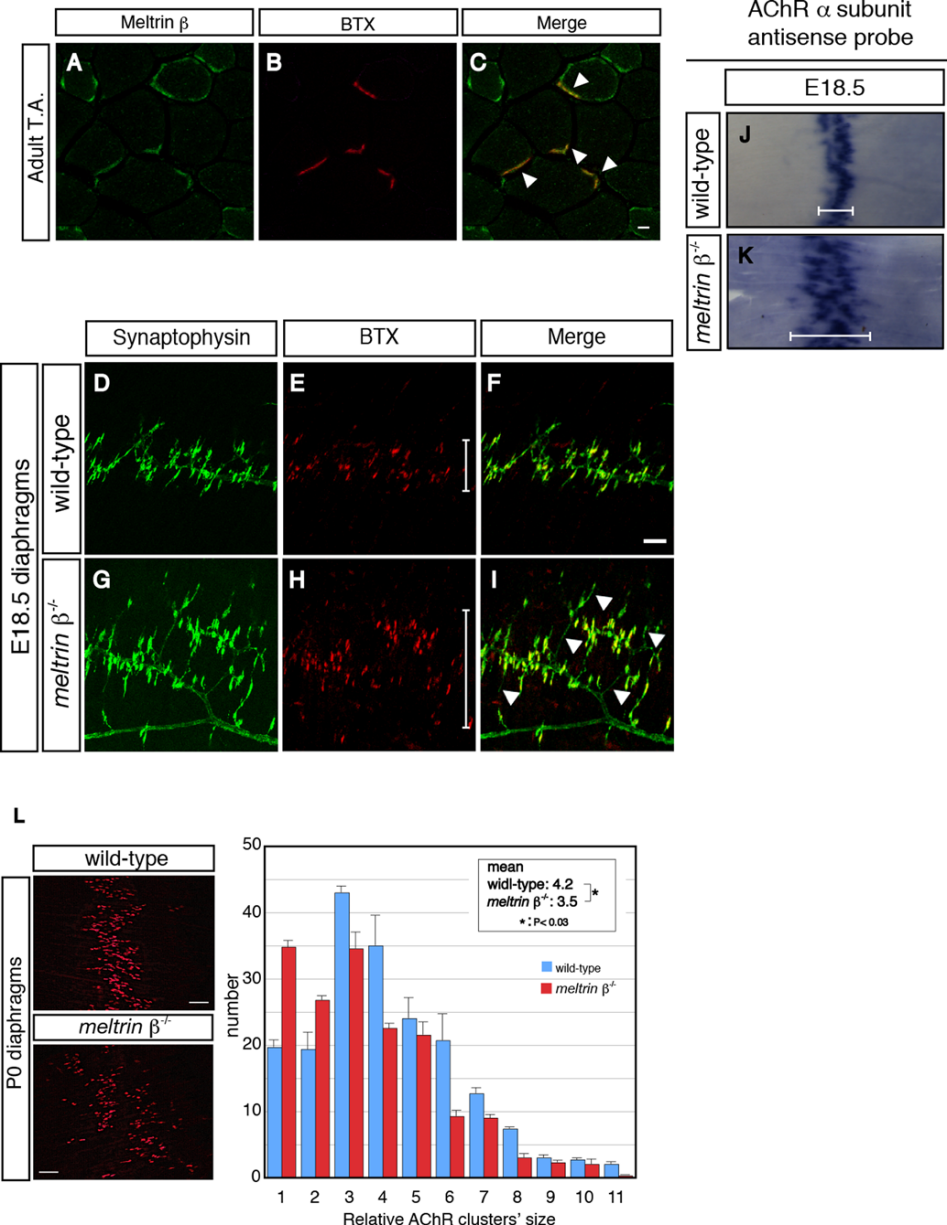
Meltrin β is expressed at the NMJ and participates in the formation of the NMJ. (A–C) Adult tibialis anterior (T.A.) muscles were stained with an anti-Meltrin β antibody (A) and Alexa-594–conjugated α-bungarotoxin (BTX) (B). Meltrin β was expressed at NMJs (C, arrowheads). Meltrin β was also expressed in a part of muscle fibers. Bar: 10 µm. (D–I) The diaphragms of wild-type (D–F) and *meltrin β* ^−/−^ (G–I) mice at E18.5 were stained with an anti-synaptophysin antibody (D and G) to label axon terminals and with BTX (E and H) to label acetylcholine receptors (AChRs). AChR clusters were distributed more broadly (H and I, also compare the bars in E and H) and the nerve terminals sprouted excessively (G and I, arrowheads) in the *meltrin β* ^−/−^ muscle compared with in the wild-type muscle (F). Bar: 100 µm. (J and K) *In situ* hybridization with probes for AChR α-subunit mRNA in E18.5 diaphragms of wild-type (J) and *meltrin β* ^−/−^ (K) mice. The central zone expressing the AChR α-subunit mRNA is broader in *meltrin β* ^−/−^ (K) than in the wild-type diaphragm (J) (compare the bars in J and K). (L) Neonatal (P0) diaphragms were stained with BTX. AChR clusters were distributed more broadly in *meltrin β^−/−^* than in wild-type diaphragms (left panel). The average size of the individual AChR clusters was smaller in *meltrin β^−/−^* muscles relative to that in wild-type muscles (right panel). Bar: 100 µm.

To examine the role of Meltrin β in the development of the NMJ, we first investigated the expression of Meltrin β at the NMJ in muscles (Fig. 1A–C). Anti-Meltrin β antibody recognizes the C-terminus of Meltrin β, and show little affinity to other proteins in *meltrin β* ^−/−^ mice ([22], also see Figure S1S). Acetylcholine receptors (AChRs) were visualized with Alexa-594–α-bungarotoxin (BTX). In the adult, Meltrin β was expressed at NMJs (Fig. 1C, arrowheads). During development, expression of Meltrin β gradually clustered at the NMJ during the late stage of embryogenesis (Figure S1).

To investigate whether Meltrin β is involved in the formation of the NMJ, we performed whole-mount immunostaining of wild-type and *meltrin β* ^−/−^ diaphragms to examine the distribution of presynaptic axon terminals and postsynaptic AChR clustering (Fig. 1D–I). In wild-type embryos at embryonic day (E) 18.5, AChR clusters were confined to the central endplate zone, and axon terminals of motor neurons detected with synaptophysin were apposed to the AChR clusters (Fig. 1D–F). In contrast, in *meltrin β* ^−/−^ embryos AChR clusters were more broadly distributed (Fig. 1H, I, also compare the bars in E and H), and excess sprouting of the axon terminals was observed prominently (Fig. 1G, I, arrowheads). Consistent with these data, the zone of AChRα mRNA distribution was broader in *meltrin β* ^−/−^ than in wild-type diaphragms at E18.5 (Fig. 1J, K; see the bars). Furthermore, distribution of AChR clusters remained sparse in diaphragms of *meltrin β^−/−^* newborns, and the average size of AChR clusters was smaller in *meltrin β* ^−/−^ than in wild-type muscles (Fig. 1L). The primary phrenic nerve trunk projected onto the muscles normally (data not shown). Together, these results indicate that Meltrin β is expressed at the NMJ and contributes to fine-tuning of the formation and/or maintenance of the NMJ.

### Gene expression pattern in synapse-rich and synapse-free sites of wild-type and *meltrin β^−/−^* muscles

**Figure 2:**
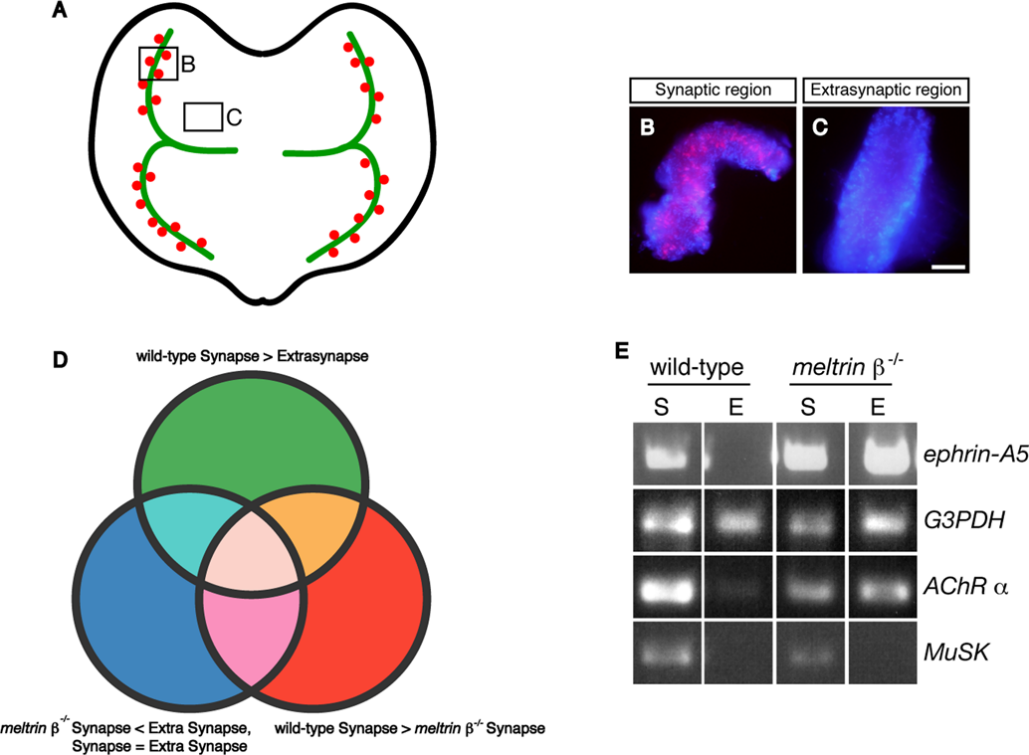
Comparison of gene expression profiles in synaptic and extrasynaptic regions of wild-type and *meltrin β^−/−^* diaphragms at E18.5. (A) Schematic diagram of synapse-rich (synaptic) (B, marked by a square) and synapse-free (extrasynaptic) (C, marked by a square) regions in the diaphragm. The primary phrenic nerve trunks and AChR clusters are indicated by green lines and red dots, respectively. (B and C) Wild-type and *meltrin β^−/−^* diaphragms at E18.5 were stained with Alexa-594–conjugated BTX (red) and DAPI (blue), and their synaptic (B) and extrasynaptic (C) regions were dissected for a microarray analysis. Bar: 100 µm. (D) All the genes expressed in the synaptic or extrasynaptic regions from wild-type and *meltrin β^−/−^* mice were classified into 3 groups that were analyzed with GCOS software. Green: genes expressed more highly in synaptic regions than in extrasynaptic regions of wild-type muscle, Red: genes expressed in synaptic regions more highly in wild-type than in *meltrin β* ^−/−^ muscle, Blue: genes expressed in extrasynaptic region more highly or expressed similarly in synaptic and extrasynaptic regions in *meltrin β^−/−^* muscles. (E) The preferential expression of the AChR α subunit and ephrin-A5 genes in synaptic regions is aberrant in *meltrin β^−/−^* diaphragm muscles at E18.5. Transcripts of several genes in synaptic (S) and extrasynaptic (E) regions were analyzed by RT-PCR. Compared with transcripts of the G3PDH gene as a control, those of AChR α subunit, MuSK, and ephrin-A5 were expressed differentially in synaptic and extrasynaptic regions in wild-type diaphragms, but the AChR α subunit and ephrin-A5 genes were expressed similarly in synaptic and extrasynaptic regions in *meltrin β* ^−/−^ diaphragms.

We hypothesized that muscles of *meltrin β^−/−^* mice would exhibit aberrant gene expression patterns related to their defects in NMJ formation. We microdissected AChR-positive “synaptic regions” (Fig. 2A, B) and AChR-negative “extrasynaptic regions” (Fig. 2A, C) from wild-type and *meltrin β* ^−/−^ diaphragms, as described previously [25]. We performed a microarray analysis, and all of the genes expressed in the synaptic and extrasynaptic regions in wild-type and *meltrin β* ^−/−^ muscles were analyzed by GeneChip Operating Software (GCOS; Affymetrix). The genes were categorized into 3 groups: genes expressed more highly in synaptic regions than in extrasynaptic regions of wild-type muscle (Fig. 2D, green), genes expressed in synaptic regions more highly in wild-type than in *meltrin β* ^−/−^ muscle (Fig. 2D, red), and genes whose expression patterns differed between wild-type and *meltrin β* ^−/−^ muscles (Fig. 2D, blue). The third class of genes included genes expressed differentially in the synaptic and extrasynaptic regions only in wild-type or *meltrin β* ^−/−^ muscles. Among 110 genes that fulfilled all the criteria (Table S1), secreted or membrane-bound proteins were selected. In addition, we sought to identify candidates that could act as a retrograde signal from the muscles to motor terminals. All of the criteria mentioned above were on the premise that the hybridization conditions for the array data from the 4 probes prepared from S (transcripts localized in the synaptic region) and E (transcripts localized in the extra-synaptic region) from wild type and *meltrin β^−/−^* muscles, respectively, were absolutely the same. However, we could not expect such ideally equal conditions of the hybridization although each array data was evaluated with several internal controls. Therefore, we next assessed several candidate genes with RT-PCR. As a result, the RT-PCR analyses revealed that *ephrin-A5*, in addition to AChR alpha, was exclusively localized in the synaptic region in wild type but not in *meltrin β^−/−^* muscle among several candidates we selected. Inconsistent with the array data, expression of ephrin-A5 was even higher in *meltrin β^−/−^* muscle than in wild type muscle. However, we were interested in this gene because of its roles in morphogenesis and synaptogenesis. A result representative of the RT-PCR analyses is shown in Fig. 2E.

### Ephrin-A5 is localized at the NMJ and participates in formation of the NMJ

**Figure 3:**
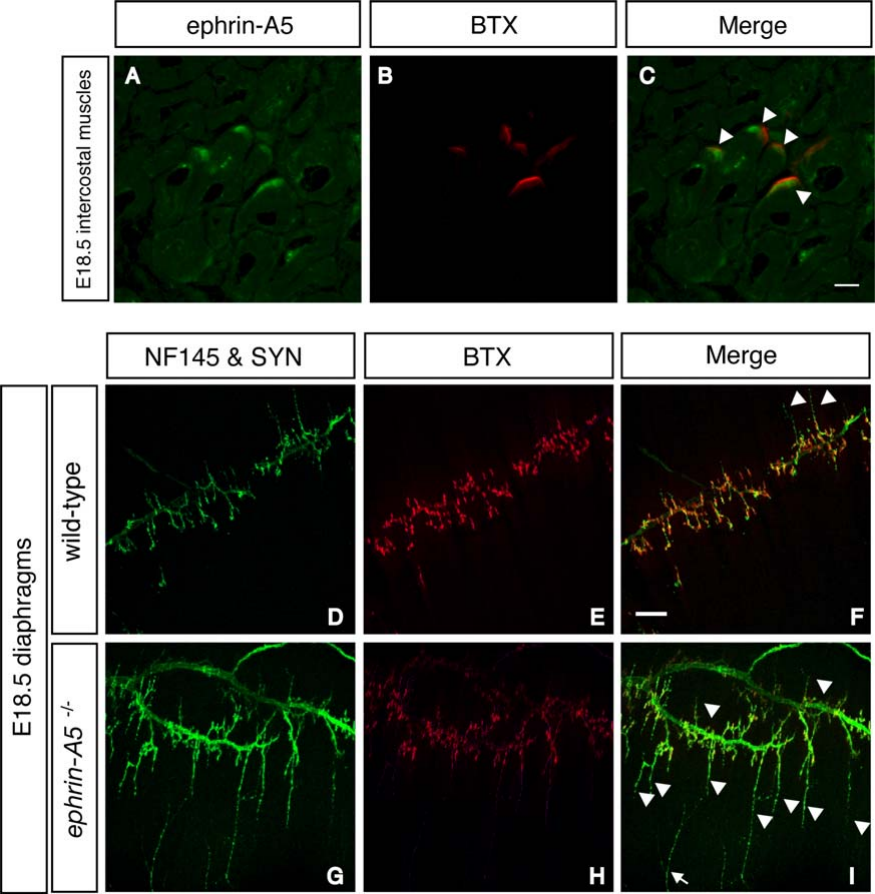
Ephrin-A5 is expressed at the NMJ during development and participates in the formation of the NMJ. (A–C) E18.5 intercostal muscles were stained with ephrin-A5 (A) and BTX (B). The BTX-positive postsynaptic apparatus colocalized with ephrin-A5 (C, arrowheads). (D–I) Diaphragm muscles from wild-type (D–F) and *ephrin-A5^−/−^* (G–I) mouse embryos at E18.5 were stained with anti-neurofilament (NF) 145 and anti-synaptophysin (SYN) antibodies (D and G) and BTX (E and H). Excess sprouting of the nerve terminals was prominent in the *ephrin-A5^−/−^* muscle (I, arrowheads. Compare with the wild-type muscle in F). Arrow: sensory neuron from the lateral part of the diaphragm. Bar: 100 µm.

Previously, Feng et al. showed that motor axons formed topographic maps on muscles and that ephrin-A5 and –A2 were involved in that topographic mapping [26] . They showed that ephrin-A5 was expressed in developing muscles. We investigated the localization of ephrin-A5 by using immunohistochemistry of E18.5 intercostal muscles (Fig. 3A–C). Ephrin-A5 protein was localized at the NMJ (Fig. 3A, C, arrowheads), a distribution that emerged gradually during the late stage of embryogenesis (Figure S1). Then, we performed whole-mount immunostaining of diaphragms from wild-type and *ephrin-A5* knockout (*ephrin-A5* ^−/−^) mice (Fig. 3D–I). In wild-type diaphragms, AChR clusters were tightly expressed in the central endplate zone opposite the nerve terminals (Fig. 3D–F). In contrast, in *ephrin-A5* ^−/−^ mice the axon terminals branched excessively (Fig. 3G–I, arrowheads). Thus, ephrin-A5 also plays a role in the precise positioning of axon terminals apposed to the postsynapses as Meltrin β does.

### Meltrin β interacts with the ephrin-A5-receptor EphA4 *in vivo*

**Figure 4:**
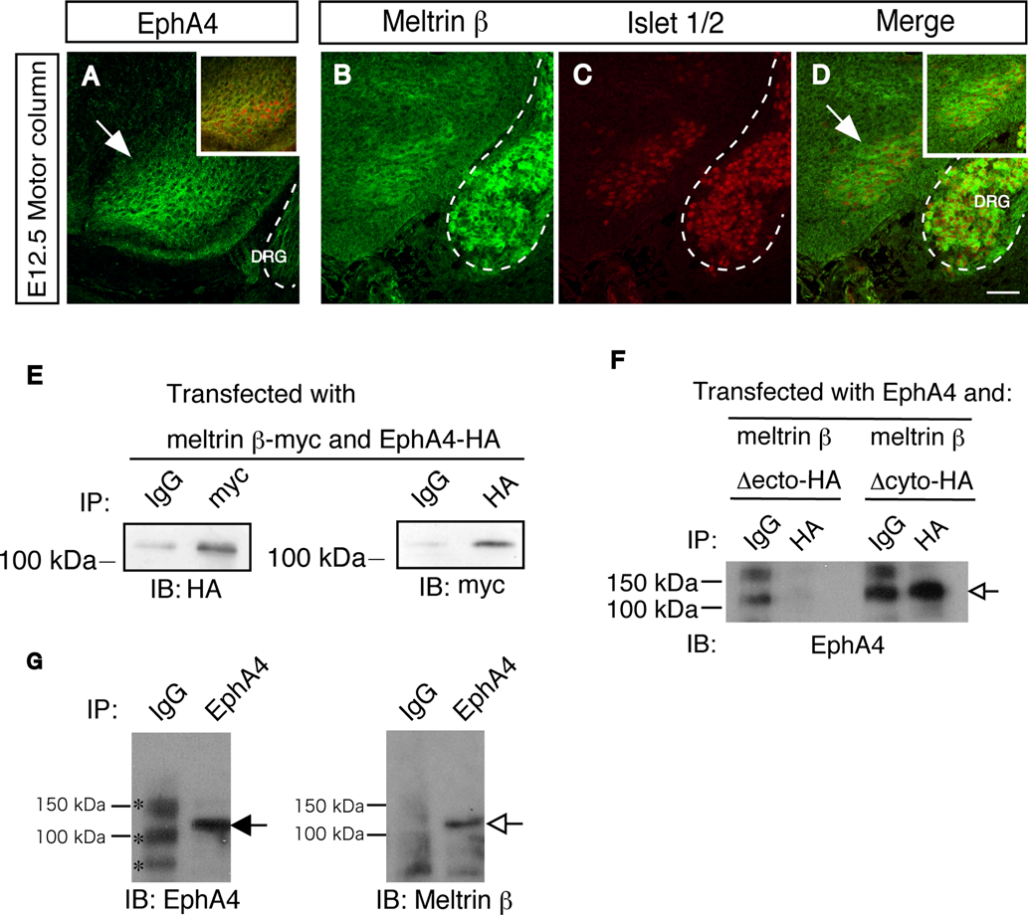
Meltrin β interacts with EphA4 in the peripheral nervous system. (A–D) Sequential sections of the spinal cord at E12.5 were double-stained with antibodies against EphA4 (A) and islet 1/2 (A, inbox) or against Meltrin β (B) and Islet 1/2 (C). EphA4 and Meltrin β proteins were expressed in Islet 1/2–positive motor neurons (indicated by arrows). Dotted lines show dorsal root ganglia (DRG). Bar: 50 µm. (E) Immunoprecipitation was performed with HEK293T cells transfected with HA-tagged EphA4 and myc-tagged Meltrin β. EphA4 coimmunoprecipitated with Meltrin β (anti-myc, left panel), and full-length Meltrin β coimmunoprecipitated with EphA4 (anti-HA, right panel). (F) Immunoprecipitation was performed with NIH3T3 cells expressing. EphA4 and *meltrin β* domain-deletion mutants tagged with HA. The ectodomain of Meltrin β, but not its cytoplasmic domain, is required for interactions with EphA4 (open arrow). (G) Coimmunoprecipitation was performed with lysates prepared from E13.5 embryonic spinal cords and DRG using the anti-EphA4 antibody used in (F). Endogenous Meltrin β (open arrow) was coimmunoprecipitated with EphA4 (closed arrow) *in vivo*. The bands for IgG and non-specific bands (asterisks) in the lane for control mouse IgG were higher than those in the lane for EphA4 because the concentration of control IgG used in this experiment was higher in this experiment, further excluding that the band for EphA4 is one of non-specific bands.

Ephrin-A5 is a glycosylphosphatidylinositol-anchored protein expressed in muscles and is a ligand for a group of membrane-bound receptor tyrosine kinases, the Ephs. Motor neurons express EphA4 receptors [27]–[30], and EphA4 is localized at the NMJ [31]. Islet 1/2–positive motor neurons of E12.5 spinal cords expressed Meltrin β as well as EphA4 (Fig. 4A–D). We asked whether Meltrin β interacts with EphA4 in motor neurons to regulate ephrin–Eph signaling. As shown in Fig. 4E–G, immunoprecipitation analyses were performed. When myc-tagged Meltrin β and HA-tagged EphA4 were co-expressed in HEK293T cells, these two proteins interacted with each other (Fig. 4E). When HA-tagged Meltrin β lacking its ectodomain was co-expressed with EphA4, the mutant Meltrin β did not precipitate EphA4; in contrast, EphA4 did co-precipitate with Meltrin β lacking the cytoplasmic domain (Fig. 4F). This suggested that Meltrin β, through its ectodomain, interacts with EphA4. We tested whether Meltrin β interacts with EphA4 in developing neural cells. We dissected spinal cords and dorsal root ganglia (DRG) from embryos at E13.5 and lysed them with Triton-X100 lysis buffer. Proteins were immunoprecipitated with the anti-EphA4 antibody and immunoblotted with the anti-Meltrin β antibody. Meltrin β co-immunoprecipitated with EphA4 (Fig. 4G, open arrow), suggesting that Meltrin β interacts with EphA4 in neural cells during development.

### Meltrin β does not affect the EphA4 expression pattern or ephrin–Eph association, but decreases the accessibility of antibodies against EphA4 C-terminus

**Figure 5:**
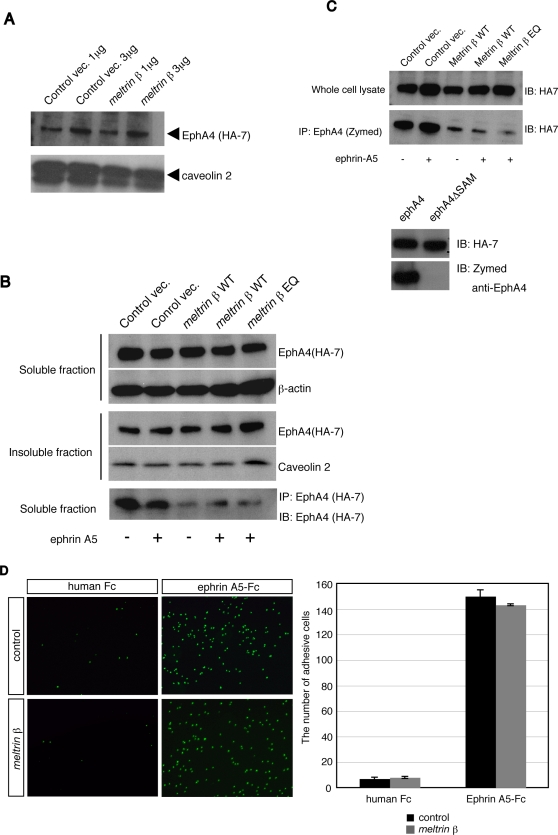
Meltrin β does not affect the expression of EphA4 on the cell surface nor the initial association between ephrin-A5 and EphA4, but decreases accessibility of antibodies that recognize the EphA4 C-terminus. (A) EphA4-HA transformant cells were transfected with Meltrin β expression plasmids (*meltrin β*) or with control vectors, and biotinylated proteins on the cell surface were precipitated with streptavidin beads. The proteins were separated by SDS-PAGE, and EphA4 was detected with an anti-HA antibody (HA-7). Caveolin 2 was used as an internal control. (B) NIH3T3 cells were transfected with EphA4-HA expression plasmids and the various plasmids listed at the top of the lanes and stimulated with ephrin-A5-Fc fusion proteins as indicated. The cell lysates were divided into a Triton-X100–solubilized fraction (soluble fraction) and Triton-X100–resistant raft microdomains (insoluble fraction). After SDS-PAGE separation, EphA4 was detected with HA-7. EphA4 proteins in the soluble fractions were also immunoprecipitated with HA-7 (the bottom panel) and detected with HA-7 after SDS-PAGE. The amount of EphA4 precipitated was reduced dramatically when the Meltrin β WT or EQ mutant was coexpressed with EphA4, regardless of stimulation with ephrin-A5. (C) (Upper Panel) NIH3T3 cells were transfected with EphA4-HA expression plasmids and the various plasmids listed at the top of the lanes and stimulated with ephrin-A5-Fc fusion proteins as indicated. EphA4 proteins in the soluble fractions (whole cell lysate) were immunoblotted with anti-HA antibody (HA-7) or immunoprecipitated with an anti-EphA4 antibody and detected with HA-7 after SDS-PAGE. Immunoprecipitation of EphA4 with anti-EphA4 was inefficient when the *meltrin β* WT or EQ mutant were coexpressed, regardless of the stimulation with ephrin-A5-Fc. (Lower Panel) An anti-EphA4 antibody from Zymed recognizes the SAM domain in the EphA4-C terminus. NIH3T3 cells were transfected with plasmids that express EphA4-HA or SAM domain–deleted EphA4-HA, and expressed proteins were detected either with HA-7 or with the anti-EphA4 antibody after SDS-PAGE of the cell lysates. (D) EphA4 transformants transfected with *meltrin β* or a control vector were seeded onto plastic dishes coated with a human Fc fragment or ephrin-A5-Fc. The binding activity of EphA4-expressing cells (detected with DAPI) on ephrin-A5-coated plates was not changed by coexpression of Meltrin β.

Meltrin β reaches lipid rafts to cleave the neuregulin-β1 extracellular region [19] , and is expressed mainly in the Golgi apparatus and the intracellular vesicles around it [20]. The molecular interaction between Meltrin β and EphA4 in neural cells led us to investigate intracellular localization of Meltrin β in the existence of EphA4. When Meltrin β was expressed together with EphA4, some of the Meltrin β proteins were translocated to the cell surface (Figure S2). Then, we examined whether Meltrin β affects the membrane localization of EphA4 on the cellular membrane. We compared the amount of EphA4 expressed on the cell surface; this was estimated by cell surface biotinylation, in NIH3T3 cells transformed with EphA4-HA, with or without the exogenous expression of Meltrin β (Fig. 5A). Meltrin β expression did not affect the expression of EphA4 on the cell surface (Fig. 5A, arrowhead). Next, we investigated the distribution of EphA4 in lipid raft and non-raft fractions (Fig. 5B). EphA4-HA was expressed together with wild-type Meltrin β (Meltrin β WT); Meltrin β EQ, which contains a point mutation in the active site of its metalloprotease domain; or the empty plasmid as a control, and total cell lysates were prepared from each transfectant with Triton-X100 lysis buffer. We divided the cellular lysates into two fractions, a Triton-X100–soluble fraction and a Triton-X100–resistant insoluble fraction, and analyzed them with anti-HA antibodies. The distribution pattern of EphA4 in the soluble and insoluble fractions was not altered by the expression of Meltrin β WT or the EQ mutant, regardless of stimulation with ephrin-A5-Fc, a chimeric protein of a soluble form of ephrin A5 and a human Fc fragment. However, the amount of EphA4 protein in the soluble fraction that was precipitated with an anti-HA antibody (clone HA-7) was reduced significantly when Meltrin β WT or the EQ mutant was expressed in the cells (Fig. 5B, bottom). Similar results were obtained with an anti-EphA4 antibody that recognizes the SAM domain in the cytoplasmic region of EphA4 (Fig. 5C). These results suggest that the C-terminus, especially SAM domain, of EphA4 becomes less accessible to these antibodies when Eph4A interacts with Meltrin β, independent of ephrin-A5 stimulation, and that the interaction of these two proteins does not require the protease activity of Meltrin β.

To investigate whether Meltrin β regulates the initial association of ephrin and Eph on the cell surface, we performed a cell adhesion assay as described previously [32]. The EphA4 transformants were seeded on plastic dishes that had been coated with human Fc fragments or ephrin-A5-Fc, and the number of the cells adhering to the dishes was counted (Fig. 5D). The number of adhesive cells was not altered by the expression of Meltrin β. Thus, expression of Meltrin β does not affect the expression pattern of EphA4 proteins in microdomains of cells or the initial association of EphA4 with ephrin-A5, although the EphA4 C-terminus becomes less accessible when Eph4A interacts with Meltrin β.

### Meltrin β regulates endocytosis of the ephrin–Eph complex

The SAM domain in the C-terminus of EphA4 is required for the endocytosis of ephrin–Eph complexes [33]. The interaction between EphA4 and Meltrin β led us to examine whether Meltrin β regulates the internalization of the ephrin-A5–EphA4 complex. Because ephrin-induced endocytosis of EphA4 is known to promote the retraction of Eph-expressing axon terminals, regulators of this endocytosis would play important roles in contact-dependent retraction during axon guidance and synaptogenesis. [34]

**Figure 6:**
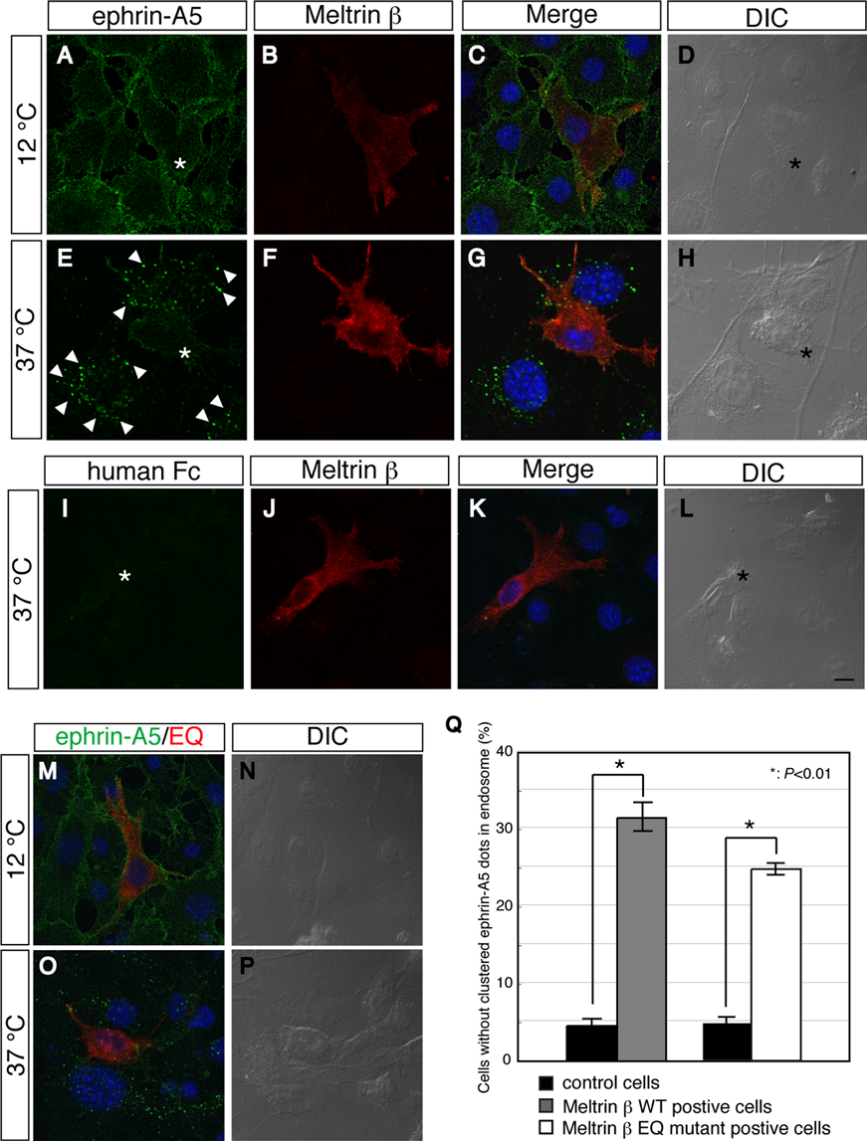
Meltrin β regulates vesicular internalization of the ephrin-A5–EphA4 complexes. (A–L) Ephrin-A5-Fc fusion proteins bound to EphA4-HA transformants equally regardless of Meltrin β expression at a temperature that inhibits endocytosis (12°C; A–D). In contrast, when the cells were put at a temperature that promotes endocytosis (37°C), Meltrin β substantially inhibited endocytosis of ephrin–Eph complexes (E–H, arrowheads: intracellular vesicles containing ephrin-Eph complexes). Control human Fc fragments do not promote endocytosis (I–L). Asterisk: Meltrin β positive cells. Blue signals in panels C, G, and K are DAPI staining of nuclei. Bar: 10 µm. (M–P) The same experiments were performed for Meltrin β EQ mutant–expressing cells. EQ mutant proteins also inhibit internalization of ephrin–Eph complexes. (Q) The number of the cells that did not have intracellular vesicles containing ephrin–Eph complexes was counted. The number of cells without endosomes containing ephrin–Eph complexes was approximately 7 times or 5 times higher in the Meltrin β WT–positive cells or in the Meltrin β EQ mutant–positive cells respectively than in the control cells (*P*<0.01, Student's *t*-test).

To test whether Meltrin β regulates the internalization of the ephrin-A5-EphA4 complex, we added ephrin-A5-Fc or human Fc fragment (as a negative control) to EphA4-HA transformants, and analyzed the complex with anti-Fc fragment antibodies by using a confocal microscope (Fig. 6). When the cells were incubated at 12°C—a process that inhibits endocytosis—ephrin-A5-Fc bound to the cell surface uniformly whether or not the cells were exogenously expressing Meltrin β (Fig. 6A–D), suggesting that Meltrin β does not affect the affinity between ephrin-A5-Fc and EphA4. When cells not transfected with Meltrin β were incubated at 37°C—a process that allows endocytotic activity—ephrin-A5-Fc was internalized into the cells after it interacted with EphA4, and numerous intracellular particles containing ephrin-Eph complexes were observed (Fig. 6E, arrowheads). In contrast, the internalization of the ephrin-Eph complexes was inhibited in cells transfected with Meltrin β, although ephrin-A5-Fc bound to the cell surface (Fig. 6E, asterisk). Human Fc fragments did not bind to or was not endocytosed significantly in this assay (Fig. 6I–L), indicating that internalization of the complexes was dependent on the association of ephrin-A5 with EphA4. As quantitative results, the number of cells without endosomes containing the ephrin-Eph complexes (that is, in which endocytosis was inhibited) was approximately 7 times higher for cells expressing Meltrin β than for control cells (Fig. 6Q). Meltrin β EQ mutant proteins also inhibited the endocytotic activity as much as Meltrin β WT did (Fig. 6M–P, Q), indicating that the protease activity of Meltrin β is not needed for this regulation of endocytosis.

## Discussion

Meltrin β is a metalloprotease-containing member of the ADAM family of proteins that is expressed in the developing heart and peripheral nervous system (PNS) [16], [17]. We previously showed that Meltrin β played a role in the endocardial cushion development. In this study, we extended the analyses of *meltrin β^−/−^* mice in the development of PNS and explored molecular mechanisms for it. Our study revealed a novel role of Meltrin β in the formation of the NMJ, and suggested a link between Meltrin β and ephrin-Eph signaling. Based on these data and previous reports, we discuss possible mechanisms through which Meltrin β contributes to the formation of NMJs together with ephrin-A5-EphA.

### Role of Meltrin β in the development of the NMJ

Muscles develop specialized postsynaptic apparatuses before motor neurons reach them [4], [6]. These prepatterned postsynaptic structures are stabilized and modified by secreted factors, such as Agrin and ACh, that emanate from motor neurons[35], [36]. In *meltrin β^−/−^* mice, AChRα mRNA was distributed in a broader zone in the muscles and excess sprouting of nerve terminals was evident. Moreover, preferential expression of the AChR α and ephrin-A5 genes in the synaptic region of muscles was disrupted in *meltrin β^−/−^* mice, whereas the expression pattern of the MuSK gene, a key player for the prepatterning of the NMJ, was not affected in these mice. These phenotypes suggest that Meltrin β does not participate in the prepatterning of the NMJ but mediates the stabilization of the NMJ through the fine-tuning of terminal axon branching and/or the transcriptional confinement of some post-synaptic genes. Consistent with this idea, Meltrin β gradually localizes to the NMJ during the late stage of embryogenesis, after the establishment of prepatterning in muscles and initial axon pathfinding although Meltrin β is expressed in the ventral horn of the spinal cord already at E12.5.

### Role of ephrin-A5 in NMJ Formation

Both Eph receptor tyrosine kinases and ephrins, high-affinity ligands of Ephs, are membrane proteins. Ephrin–Eph signals function as topographical guidance cues for neurons [26], [29], [37] and synaptogenesis and plasticity of synapses in the central nervous system [38]. Ephrin-A5 is expressed in the developing muscles [26] and our study revealed that ephrin-A5 proteins were concentrated at the NMJ (Fig. 3). Other ephrin-As and EphAs, including ephrin-A2, EphA4, and EphA7, also localize at the NMJ in adult or postnatal muscles [31]. The localized expression of these ephrins and Ephs at the NMJ suggests that they also function at the interface between motor neurons and muscles during late stages of development and/or in adult for the formation or maintenance of the NMJ.

Previous studies showed that Ephrin-A5 is a rostrocaudal positioning cue for the projection of some motor neurons [26] and a guidance cue for another group of motor neurons innervating the muscles of the dorsal limb [28], [30]. We asked whether *ephrin-A5* was also involved in the fine- tuning of motor axon terminals during development. In *ephrin-A5* ^−/−^ embryos, axon arbors failed to be stabilized at the postsynaptic sites and excess sprouting of the motor terminals was prominent at E18.5 in *ephrin-A5* ^−/−^ mice. This phenotype was similar to, but more pronounced than, that of *meltrin β^−/−^* mice. These results indicate that ephrin-A5 is required for the axon terminals to contact with postsynaptic apparatuses properly. *Ephrin-A5* ^−/−^ mice survive to adulthood. The NMJ of their muscles after birth would be also worth studying in detail because it is possible that signs of abnormality in the NMJ of these mice have been unnoticed. Alternatively, because structurally similar ephrins are expressed in muscles, they might compensate the phenotype after birth.

In contrast to the molecular mechanisms of prepatterning of the NMJ postsynaptic regions, the factors produced by muscles to differentiate presynaptic specializations have been poorly understood. The interaction of ephrins with Ephs leads to bidirectional signaling events [39], [40], and ephrin–Eph interactions can induce attractive or adhesive responses in addition to repulsive effects [40]–[42]. Whereas the forward signaling from ephrin-A5 to EphA4 causes cytoskeletal rearrangements in some conditions [39], [40], the reverse signal from EphA to ephrin-A5 upregulates the expression of integrin-β1 [43] to modify cell morphology. Integrin-β1 in muscles is essential for the formation of stable NMJs [44], supporting a molecular link between ephrin-A5 and integrin-β1 in NMJ formation. In Xenopus, ephrin-A5 acts as an attractive signal to neurons in cooperation with laminin [45]. At the NMJ, a specific laminin, laminin-β2 [46], [47], may cooperate with ephrin-A5 to attract and stabilize motor terminals. Thus, the adhesive ephrin–Eph interaction at the NMJ could be a noticeable mechanism through which contact-dependent bidirectional signaling contributes to development of the pre- and postsynaptic specializations.

### A regulatory role for Meltrin β in ephrin-A5–EphA signaling

The molecular interaction between Meltrin β and EphA4, the similarity of the phenotypes of *ephrin-A5* ^−/−^ and *meltrin β^−/−^* mice at the NMJ, and the aberrant ephrin-A5 mRNA expression in *meltrin β^−/−^* mice suggest a relationship between Meltrin β and ephrin-A5–EphA signaling. Although Meltrin β was not found at the cell surface without coexpressing with EphA4, some Meltrin β molecules were translocated to the cell surface when Meltrin β was co-expressed with EphA4 (Figure S2). Because Meltrin β is not usually found at the cell surface, we interpret the result as indicating that the molecular interaction with EphA4 likely promoted the translocation of Meltrin β to the cell surface.

The most important question was whether Meltrin β regulates the ephrin-A5–EphA4 interaction. An ephrin–Eph interaction delivers a contact-triggered repulsive signal to the ephrin- and Eph-expressing cell [11], [39]. Mechanisms to convert the signal from contact to repulsion include cleavage of the ephrin. Ephrin-A2 is cleaved by ADAM10 *in cis* upon binding to EphA receptor, and a cleavage-inhibiting mutation in ephrin-A2 delays axon withdrawal [11]. In contrast, Janes et al. showed that ADAM10 cleaves ephrin-A5 on the membranes of opposing cells (*in trans*; [12]). Either result of these experiments with cultured cells can explain contact-triggered repulsion between ephrin- and Eph-expressing cells. Another mechanism is endocytosis. Ephrins or Ephs on opposing cells are endocytosed into the cells expressing Ephs or ephrin, respectively, without ectodomain shedding, a process called trans-endocytosis [34], [48]–[50]. With some evidences, it is also suggested that endocytosis is a critical step for turning the interaction of ephrins and Ephs from contact into repulsion efficiently [34].

In contrast to ADAM10, Meltrin β did not cleave ephrin-A5 even when ephrin-A5–expressing cells were mixed with EphA4-expressing cells (data not shown). Instead, the expression of Meltrin β in EphA4-expressing cells interfered with the endocytosis of ephrin-A5–EphA4 complexes (Fig. 6). This interference might diminish the ability of ephrin- and Eph-expressing cells to repel with each other. Alternatively, cell-cell adhesion would overcome the repulsion when EphA4 interactes with Meltrin β.

How does Meltrin β regulate the endocytosis of ephrin-A5–EphA4 complexes? Endocytosis is triggered by Vav family GEFs, which bind to phosphorylated Ephs and are themselves phosphorylated by other kinases, possibly of the Src family [51], [52]. This results in the activation of Rac1 GTPase and the endocytosis of ephrin–Eph complexes [34]. In the current study, when EphA4 interacted with Meltrin β, EphA4 became less accessible to an antibody against its SAM domain significantly. We speculate that the association of EphA4 with Meltrin β may alter the accessibility of endocytosis regulators to EphA4 or change their association and may prevent Vav family GEFs from activating ephrin-triggered endocytosis.

**Figure 7:**
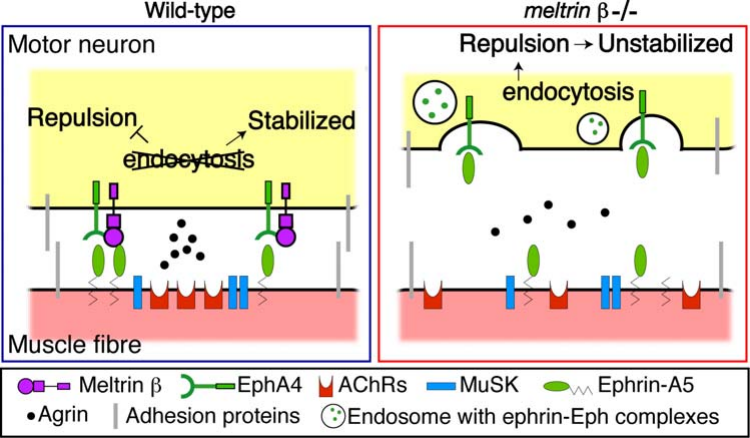
Hypothetic model of how Meltrin β and ephrin-A5–EphA4 signaling stabilize the NMJ. When axon terminals touch the ephrin-A5-expressing postsynaptic region, EphA4 associated with Meltrin β is localized preferentially at the axon terminal opposite the postsynaptic cluster on the muscle fiber; Meltrin β interferes with the internalization of the ephrin-A5–EphA4 complexes so that the axon terminal and muscle could avoid receiving a repulsive signal at the NMJ, and many molecules, such as agrin, MuSK and adhesion proteins, cooperate in stabilization of the NMJ. In the absence of Meltrin β, axon terminals of motor neurons cannot be stabilized because impaired suppression of endocytosis would result in a failure to block the ephrin-A5–EphA4 repulsive signal. As a result, the motor terminals remain mobile at the NMJ, and the reverse signal from EphA4 to ephrin-A5 is unstable, leading to the diffuse distribution of ephrin-A5 and AChR transcripts in muscles. (see also text).

On the basis of our findings and previous reports, we present a working hypothesis of how Meltrin β and ephrin-A5–EphA signaling cooperate to stabilize the association of the pre- and postsynaptic apparatuses of the NMJ (Fig. 7). In short, Meltrin β associated with EphA4 would convert a state of an axon terminal from a mobile to a static one through modulating the ephrinA5-EphA4 signaling at the NMJ by interfering with the internalization of the ephrin-Eph complexes there. This conversion might contribute to formation of the stabilized NMJ. The sustained expression of EphA4-Meltrin β at the presynapses would cause localized expression of ephrin-A5 at the postsynapses, which would increase the stability of the ephrin-A5-EphA4 interaction.

In summary, this study revealed that Meltrin β plays a role in the fine tuning of the NMJ during development. Although we discussed a non-proteolytic role of Meltrin β in the regulation of ephrin-A5-EphA4 signaling here, functional significance of Meltrin β as a protease is not excluded in the NMJ formation. Further studies are necessary to determine whether Meltrin β presented together with EphA4 on the cell surface also functions as a protease to cleave substrates, which contribute to the formation of the NMJ including the confinement of AChR mRNA in the central endplate zone.

In addition to the question on the proteolytic function of Meltrin β, many questions remain to be solved: the molecular mechanisms by which Meltrin β blocks the ephrin-dependent endocytosis and the mechanisms that localize the Meltrin β–EphA4 complex in axon terminals apposed to the NMJ. Meltrin β and EphA4 are not only expressed in neurons but also in developing muscles (this study and [31]), and ephrin-A5 could be also expressed in terminal Schwann cells. Cell type-specific ablation of Meltrin β and ephrin-A5 would help understanding more precise mechanisms of the NMJ stabilization which these molecules mediate. It is noteworthy that the expression of ephrins, Ephs, and Meltrin β in the NMJ is sustained in adult mice, implying that these molecules are also important in the maintenance of the NMJ.

## Materials and Methods

### Generation of meltrin *β* ^−/−^ and *ephrin-A5^−/−^* mice

All procedures with experimental animals were approved by the Institutional Animal Care Research Advisory Committee at Kyoto University. C57BL/6 *meltrin β* ^−/−^ mice, in which the active site of the metalloprotease domain was deleted, were generated previously [22], and *ephrin-A5^−/−^* mice were generated as described previously [37].

### Antibodies

The following antibodies were used: anti-NF145 (polyclonal; Chemicon), anti-synaptophysin (polyclonal; Zymed), anti-hemagglutinin (HA clone HA-7; Sigma), anti-EphA4 (polyclonal: Santa Cruz Biotechnology Inc.; clone 4C8H5: Zymed), anti-Meltrin β (developed by our lab as described previously [18]), anti–islet 1/2 (monoclonal; Developmental Studies Hybridoma Bank), anti-ephrin-A5 (polyclonal; R&D Systems), anti-β-actin (clone AC-74; Sigma), anti-caveolin 2 (clone 65; BD Transduction Laboratories), and anti-human Fc fragment (polyclonal; Jackson ImmunoResearch).

### Plasmids

The following expression plasmids or control plasmids were used: pCMV-SPORT 6-*ephA4* (Invitrogen), pcDNA3.1-*ephA4*-*HA* (the cytoplasmic end of *ephA4* was replaced with a HA tag in the pcDAN3.1 expression plasmid), pcDNA3.1-*ephA4*ΔSAM-HA (the SAM domain of *ephA4* was deleted followed by its endogenous PDZ-binding sequence), pBIE-*meltrin β* Δectodomain-HA (the *meltrin β* ectodomain was replaced by HA in the pBIE expression plasmid, [18]), pBIE-*meltrin β* Δcytoplasmic domain-HA (the *meltrin β* cytoplasmic domain was replaced by HA in the pBIE expression plasmid), pBOS, pBOS-*meltrin β* wild-type (WT), and the pBOS-*meltrin β* EQ mutant (mutation in the metalloprotease active site to delete metalloprotease activity).

### Immunohistochemistry for sections and whole-mount diaphragms

T.A. muscles and intercostal muscles were dissected from wild-type adults and E18.5 embryos, respectively, and frozen immediately. Sections were cut at 10 µm and fixed with ice-cold acetone for 10 minutes. They were used for immunohistochemistry with an anti-Meltrin b and anti-ephrin-A5 antibodies. The signal was amplified with the TSA biotin system (PerkinElmer). For immunofluorescent staining of the embryonic spinal cords, E12.5 embryos were dissected and fixed with 4% PFA. After 3 washes in PBS, the solution was replaced with 30% sucrose in PBS. The embryos were sectioned at 14 µm, and immunofluorescence was performed as described previously [30]. For the analyses of the NMJ, the heads were removed from E18.5 embryos or postnatal day (P) 0 newborns and the bodies were fixed in 4% paraformaldehyde (PFA) overnight at 4°C, washed twice with phosphate buffered saline (PBS), and whole mount immunostaining were performed. Alexa-594–conjugated BTX (Molecular Probes) was used to label AChRs.

To assess the distribution of the size of AChR clusters in P0 diaphragms, Z-stack confocal images were captured on a Leica TCS SP5 confocal microscope (Leica Microsystems), and Image J software was used to divide clusters into 11 classes according to their pixel size.

### Microarray analysis

The BTX-positive synapse-rich (synaptic) and synapse-free (extrasynaptic) regions of the diaphragms from E18.5 embryos were obtained as described previously [25]. Each region was taken from 3 wild-type and 3 *meltrin β^−/−^* mouse embryos. Biotinylated RNA probes were synthesized according to the instructions of the GeneChip manufacturer (Affymetrix). The chips were analyzed with the GCOS analysis system.

### Whole-mount diaphragm *in situ* hybridiation

The E18.5 diaphragms were fixed in 4% PFA and dehydrated in 100% methanol. After rehydration, the endogenous peroxidase was inactivated with 6% H_2_O_2_. An antisense riboprobe for the AChRα subunit was synthesized with a DIG-labeling kit (Roche). Hybridization and color development were done by a standard procedure [8].

### Reverse transcription – polymerase chain reaction (RT-PCR)

Total RNA preparations were obtained from the synaptic and extrasynaptic regions from E18.5 diaphragms of wild-type and *meltrin β^−/−^* mice. Primers: AChR a subunit, sense; CACTTTCCCTTCGATGAGCAGA, anti-sense; CCACAATGACCAGAAGGAACAC, MuSK, sense; CAACATTCCCGTCAATAACGTC, anti-sense; GTTTCACAATGTTGGGGTTGTC, ephrin-A5, sense; ATGTTGACGCTGCTCTTTCTGGT, anti-sense; CGTTGTCTGGGATTGCAGAGGA.

### Coimmunoprecipitation assay *in vitro* and *in vivo*

E13.5 spinal cords and DRG dissected and lysed with Triton-X100 lysis buffer (25 mM Tris-HCl [pH 7.5], 100 mM NaCl, 2 mM EDTA, and 1% Triton-X100) containing protease inhibitor cocktail (Nacalai, Japan) and incubated on ice for 1 h. The lysate was centrifuged at 14,000 rpm for 10 minutes at 4C, and the supernatant was precleared with protein G–conjugated Sepharose beads (GE Healthcare) for 1 h at 4C, followed by incubation with the anti-EphA4 antibody (Zymed) or control mouse IgG overnight at 4C. Protein G-conjugated Sepharose beads were added and washed several times after centrifugation, and then SDS-PAGE was performed.

HEK293T cells or NIH3T3 cells were transfected with plasmids by using the FuGene HD transfection reagent (Roche). The cells were incubated for 36 h and then washed with ice-cold PBS and lysed with Triton-X100 lysis buffer containing proteinase inhibitor cocktail. Immunoprecipitation was performed as described above.

### Endocytosis assay

EphA4-HA transformant cells were transfected with pBOS-*meltrin β* WT or EQ and incubated for 36 h, followed by replacement of the growth medium with serum-free medium for 2 h. The cells were washed once with Hanks balanced salt solution (HBSS, Invitrogen) and incubated in HBSS at 12°C or 37°C for 10 min. After this treatment, 3 µg/ml human Fc or ephrin-A5-Fc (R&D systems, nonclustered) proteins were added, and the mixture was incubated for 15 min at 12°C or 37°C respectively. The cells were washed twice with HBSS and fixed with 4% PFA, followed by permeabilization with 0.2% Triton-X 100/PBS solution. To label human Fc or ephrin-A5-Fc proteins, goat anti-human Fc fragment antibodies were added before the permeabilization for the cells incubated at 12°C and after the permeabilization for the cells incubated at 37°C. Immunocytochemistry was performed. Single sections of confocal images were captured on a Leica TCS SP5 confocal microscope. Several fields were chosen randomly and the number of cells without endosomes containing ephrin–Eph complexes was counted. The experiment was performed in three 2-well glass culture slides on two independent occasions. Student's *t-test* was used for statistical comparisons.

## Supporting Information

Figure S1 (A–I) Meltrin β was not detected at the NMJ (BTX) at E16.5 (A–C) but was clearly clustered at E18.5 (D–F: arrowheads). The expression of Meltrin β was sustained in adult muscles (G–I: arrowheads; also shown in Fig. 1). (J–R) Ephrin-A5 was not detected at the NMJ at E16.5 (J–L) but was clearly clustered at E18.5 (M–O: arrowheads; also shown in Fig. 3). Expression of ephrin-A5 was sustained in adult muscles (P–R: arrowheads). (S) Anti-Meltrin β antibody recognized Meltrin β proteins specifically at the NMJ in E18.5 intercostal muscles. Meltrin β signal could be hardly detected in meltrin β−/− intercostal muscles. (T and U) Negative controls for immunostaining: rabbit IgGs were used instead of the anti-Meltrin β antibody (T and T'), and goat IgGs were used instead of the anti-ephrin-A5 antibody (U and U').
(2.69 MB TIF)

Figure S2 (A and B) NIH3T3 celles were transfected with only Meltrin β, cultured for 24 hours and immunocytochemistry was performed with anti-Meltrin β antibody. Most of the Meltrin β proteins were localized in cytoplasmic region as described previoulsly. Bar: 10 µm. (C–E) NIH3T3 cells were transfected with EphA4 and Meltrin β and cultured for 24 hours. The cells were fixed, and Meltrin β and EphA4 were detected with anti-Meltrin β antibody and ephrin-A5-Fc respectively. Ephrin-A5-Fc-bound EphA4 proteins were localized on the plasma membrane (C and E). Meltrin β was also detected on the plasma membrane (D and E), although most Meltrin β proteins were localized in the endoplasmic reticulum and the Golgi apparatus. (C'–E') Magnifications of the square in C are shown. Meltrin β colocalized with EphA4 (arrowheads). Upper bar: 10 µm, lower bar: 5 µm.
(0.68 MB TIF)

Table S1 The expression profiles in the synaptic and extrasynaptic regions in wild-type and meltrin β−/− diaphragms were compared by microarray analysis. All expressed genes were divided into 3 groups: genes expressed more highly in synaptic regions than in extrasynaptic regions of wild-type muscle; genes expressed in synaptic regions more highly in wild-type than in meltrin β−/− muscle; and genes whose expression pattern was different between wild-type and meltrin β−/− muscles, including genes expressed differentially in the synaptic and extrasynaptic regions only in wild-type or meltrin β−/− muscles. Genes that satisfied the criteria of all 3 categories (represented by the light pink region of overlap in Fig. 2D) are listed in this table.
(0.97 MB TIF)
