# Supplementary figures and images for: Meltrin β/ADAM19 Interacting with EphA4 in Developing Neural Cells Participates in Formation of the Neuromuscular Junction
Source: PLoS One. 2008 Oct 2;3(10):e3322. doi: 10.1371/journal.pone.0003322 (PMC2552171; doi:10.1371/journal.pone.0003322)

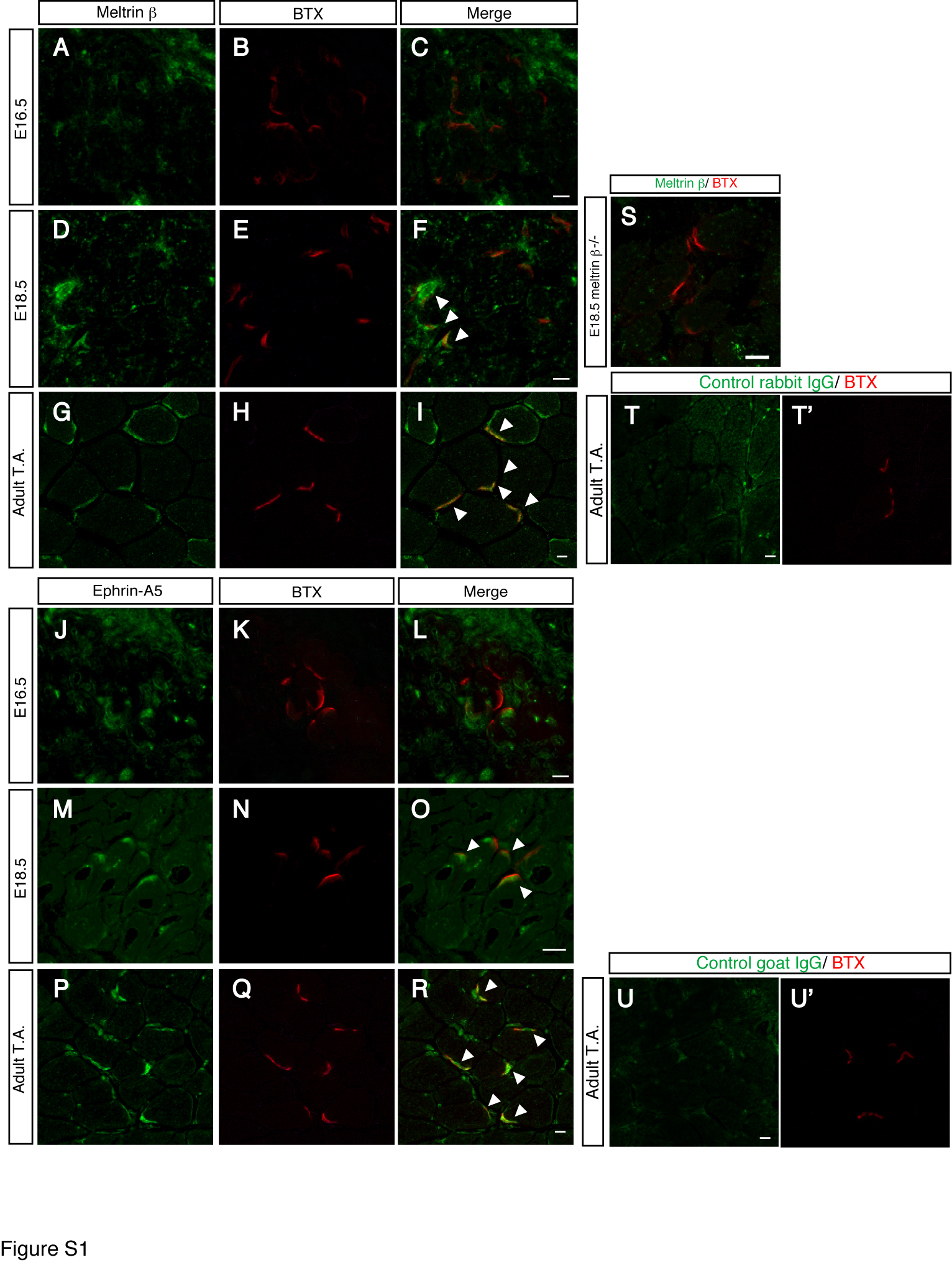

Supplement: Figure S1 — (A–I) Meltrin β was not detected at the NMJ (BTX) at E16.5 (A–C) but was clearly clustered at E18.5 (D–F: arrowheads). The expression of Meltrin β was sustained in adult muscles (G–I: arrowheads; also shown in Fig. 1). (J–R) Ephrin-A5 was not detected at the NMJ at E16.5 (J–L) but was clearly clustered at E18.5 (M–O: arrowheads; also shown in Fig. 3). Expression of ephrin-A5 was sustained in adult muscles (P–R: arrowheads). (S) Anti-Meltrin β antibody recognized Meltrin β proteins specifically at the NMJ in E18.5 intercostal muscles. Meltrin β signal could be hardly detected in meltrin β−/− intercostal muscles. (T and U) Negative controls for immunostaining: rabbit IgGs were used instead of the anti-Meltrin β antibody (T and T'), and goat IgGs were used instead of the anti-ephrin-A5 antibody (U and U'). (2.69 MB TIF) [file pone.0003322.s001.tif]

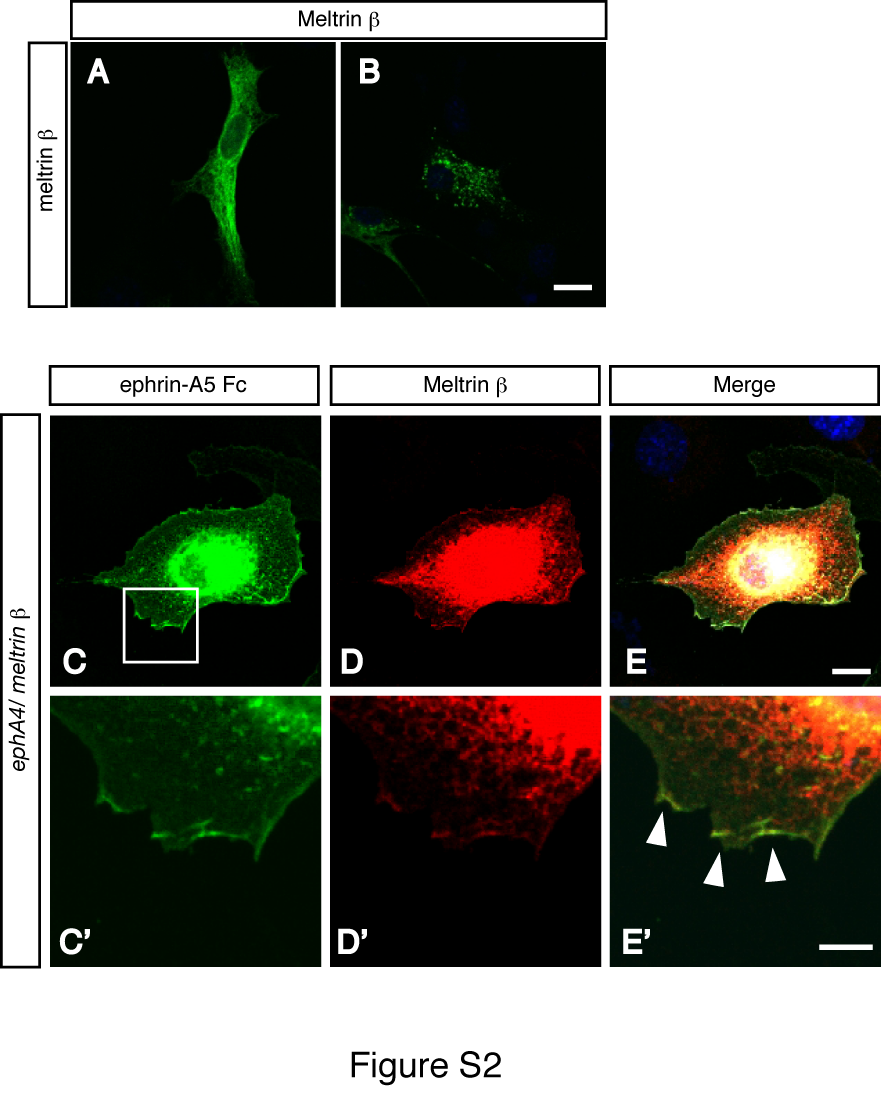

Supplement: Figure S2 — (A and B) NIH3T3 celles were transfected with only Meltrin β, cultured for 24 hours and immunocytochemistry was performed with anti-Meltrin β antibody. Most of the Meltrin β proteins were localized in cytoplasmic region as described previoulsly. Bar: 10 µm. (C–E) NIH3T3 cells were transfected with EphA4 and Meltrin β and cultured for 24 hours. The cells were fixed, and Meltrin β and EphA4 were detected with anti-Meltrin β antibody and ephrin-A5-Fc respectively. Ephrin-A5-Fc-bound EphA4 proteins were localized on the plasma membrane (C and E). Meltrin β was also detected on the plasma membrane (D and E), although most Meltrin β proteins were localized in the endoplasmic reticulum and the Golgi apparatus. (C'–E') Magnifications of the square in C are shown. Meltrin β colocalized with EphA4 (arrowheads). Upper bar: 10 µm, lower bar: 5 µm. (0.68 MB TIF) [file pone.0003322.s002.tif]

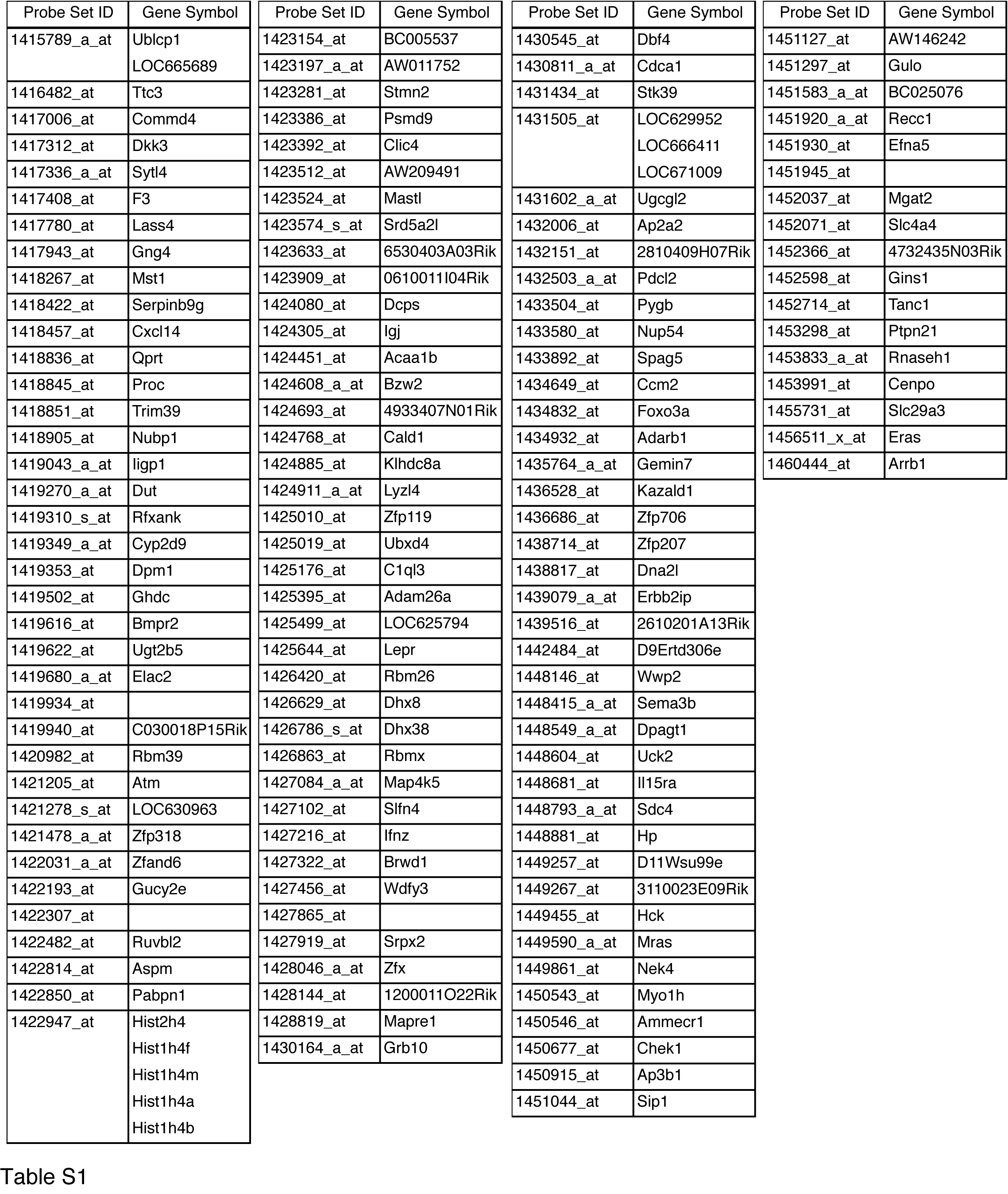

Supplement: Table S1 — The expression profiles in the synaptic and extrasynaptic regions in wild-type and meltrin β−/− diaphragms were compared by microarray analysis. All expressed genes were divided into 3 groups: genes expressed more highly in synaptic regions than in extrasynaptic regions of wild-type muscle; genes expressed in synaptic regions more highly in wild-type than in meltrin β−/− muscle; and genes whose expression pattern was different between wild-type and meltrin β−/− muscles, including genes expressed differentially in the synaptic and extrasynaptic regions only in wild-type or meltrin β−/− muscles. Genes that satisfied the criteria of all 3 categories (represented by the light pink region of overlap in Fig. 2D) are listed in this table. (0.97 MB TIF) [file pone.0003322.s003.tif]
